# Diagnosis and management of a rare bilateral ovarian mixed germ cell tumor: a case report

**DOI:** 10.3389/fonc.2025.1504231

**Published:** 2025-09-12

**Authors:** Xuanling Li, Min You, Xiaoyun Zhang, Jingjing Wei, Guangyao Lin, Qianjue Tang, Lianwei Xu

**Affiliations:** ^1^ Department of Gynecology, Longhua Hospital, Shanghai University of Traditional Chinese Medicine, Shanghai, China; ^2^ Department of Pathology, Longhua Hospital, Shanghai University of Traditional Chinese Medicine, Shanghai, China

**Keywords:** diagnosis and management, four-component, ovarian mixed germ cell tumor, dysgerminoma, case report

## Abstract

**Background:**

Mixed ovarian malignant germ cell tumors (MOGCTs) are rare neoplasms composed of two or more malignant germ cell components, representing less than 1% of all ovarian germ cell tumors. They primarily affect adolescents and young women, presenting a clinical challenge due to their histologic heterogeneity, potential for recurrence, and the need to balance oncologic safety with fertility preservation.

**Case presentation:**

We reported a 22-year-old woman diagnosed with a four-component MOGCT in the right ovary—comprising yolk sac tumor, immature teratoma, embryonal carcinoma, and dysgerminoma—along with a dysgerminoma component in the left ovary. Considering her age and fertility desire, fertility-sparing surgery was performed, followed by adjuvant BEP chemotherapy. At 12-month follow-up, the patient remained disease-free with regular menstruation and no signs of recurrence.

**Conclusion:**

This case highlights the feasibility of fertility-sparing treatment in patients with complex bilateral MOGCTs. Given the rarity and histological diversity of such tumors, individualized treatment planning, strict staging, and long-term surveillance are essential to optimize clinical outcomes and preserve reproductive potential.

## Introduction

MOGCT is a histological heterogeneous tumor originating from embryonic gonadal germ cells. Malignant ovarian germ cell tumors (MOGCT) account for only 2 - 5% of all ovarian malignancies (1). The typical clinical manifestations of MOGCT are palpable masses and/or abdominal distension, lower abdominal pain, and urinary system symptoms among others. Most MOGCTs are diagnosed when limited to the ovaries (stage I), and these early tumors can be treated separately through surgery. The most common histological subtypes of MOGCT include dysgerminomas, immature teratomas, yolk sac tumors, and mixed germ cell tumors, while other less common MOGCTs include, choriocarcinoma, and ovarian choriocarcinoma (2). Yolk sac tumor is a rare malignant ovarian tumor, comprising less than 1% of all malignant germ cell tumors, and more common among teenagers and young women (3). Ovarian dysgerminomas (OD) is a medium to low-grade malignant tumor formed by abnormal proliferation of primordial germ cells. Most dysgerminomas are unilateral, with only 15% to 20% being bilateral (4), mainly occurs in young people aged 20 - 30, and is more invasive than dysgerminomas, making it a highly malignant tumor, and immature teratoma is a malignant tumor composed of three layers of germ cells, its recurrence and metastasis are directly related to the quantity and immaturity of the contained neuroepithelium (5). Due to the rarity of these malignant tumors, clinical trial data is severely lacking and their progression lags behind other tumors, so more case studies are needed to support discussion. Surgical staging is the first step in MOGCT management. Currently, for early patients with fertility requirements, surgery that preserves reproductive function is preferred. However, the necessity and scope of comprehensive staged surgery for early MOGCT patients are still controversial (6).

In 2009, a multidisciplinary team consisting of gynecological oncologists, pediatric oncologists, and pediatric surgeons studied this type of tumor under the guidance of the International Alliance for Malignant Reproductive Cells (MaGRC). Important organizations such as the European Society of Gynecological Oncology (ESGO) and the European Society of Pediatric Oncology (SIOPE) have established diagnostic criteria, treatment, and follow-up (7). Currently, multiple studies have confirmed the safety and effectiveness of preserving reproductive function in MOGCT patients. The earliest related study was conducted by Kurman and Norris in 1977, and no difference in prognosis was observed in a cohort of 182 early MOGCT patients who underwent conservation surgery (8). In the following 50 years, the tremendous development of surgery and chemotherapy allowed women with this malignant tumor to retain their fertility and be completely cured. Literature reports suggest a nearly 100% 5-year survival rate in dysgerminoma cases, while the 5-year survival rate of women with non-asexual germ cell tumors is 85%. Among them, embryonic cancer has the worst prognosis, with a total 5-year survival rate of only 33.3% (9). This indicates that fertility sparing surgery (FSS) (unilateral salpingo-oophorectomy and uterine preservation) is not only feasible and safe in the early stages of the disease, but also in the late stages. To our knowledge, bilateral ovarian germ cell tumors are relatively rare, and unilateral mixed germ cell tumors combined with contralateral germ cell tumors are even rarer. This article reported a 22-year-old patient with mixed germ cell tumors, including yolk sac tumors, immature teratomas, embryonic cancers, and asexual cell tumors, accompanied by contralateral asexual cell tumors. We performed unilateral adnexectomy, contralateral cyst removal, and integrated traditional Chinese. After surgery, BEP chemotherapy was administered. Currently, 12 months after surgery, the menstrual cycle has recovered well, the condition is stable, and no tumor recurrence has been observed. Given the rarity of this disease, it is crucial to strictly stage treatment and establish personalized postoperative monitoring before more effective evidence-based guidance appears. Our study aims to report a case of MOGCT patient and explore the significance of preserving the patient’s uterus and the mildly diseased ovary to maintain fertility and improve their prognostic value, in order to provide some reference for clinical diagnosis and treatment of patients with bilateral ovarian malignant tumors.

## Case presentation

A 22-year-old nulliparous woman with regular menstrual cycles presented with a 4-day history of right lower abdominal and lumbar pain. Initial abdominal ultrasound on July 14, 2023, revealed a slightly hyperechoic mass in the right adnexal region. Pelvic MRI confirmed a solid right adnexal mass measuring 14 × 9.2 × 9.8 cm and a cystic lesion in the left adnexa. Serum tumor markers were elevated, with AFP exceeding 1000 ng/mL and β-HCG at 401.35 mIU/mL. On July 18, 2023, the patient was admitted to our hospital for treatment. Upon examination, the abdomen was found to be swollen, and a lump was palpated in the middle and lower abdomen up to three fingers above the navel. Laboratory tests showed markedly elevated AFP (20,233 ng/mL), β-HCG (658.64 mIU/mL), NSE (20.4 ng/mL), and HE4 (72.6 pmol/L), while other tumor markers remained within normal limits. PET-CT demonstrated a large pelvic mass with heterogeneous FDG uptake, suggestive of a malignant germ cell tumor arising from the right adnexa. Mild FDG uptake was also observed in retroperitoneal and bilateral iliac lymph nodes, as well as along the wall of a left adnexal cyst. No abnormal FDG accumulation was detected elsewhere, including the brain.

**Figure 1:**
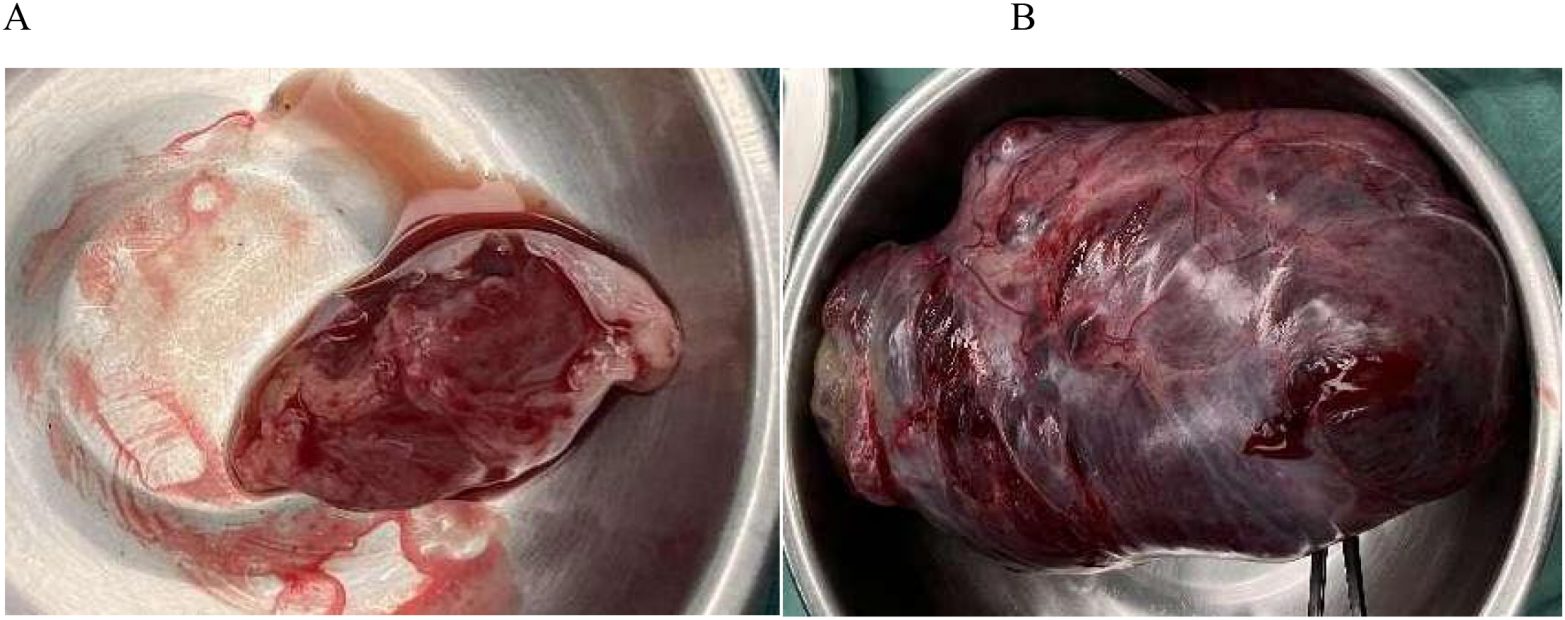
Giant pelvic tumor **(A)**; Left ovarian cyst **(B)**; Giant mass on the right side.

Before surgery, we discussed fertility preservation with the patient and their family. Despite the malignant diagnosis, they expressed a strong desire to preserve fertility and understood the potential need for subsequent chemotherapy. Surgical treatment was performed on July 24, 2023. During the operation, a small amount of ascites of about 50ml was observed, and no malignant tumor cells were found in the ascites during the operation; No obvious abnormalities were found in the appearance of liver, gallbladder, pancreas, spleen, kidney, and greater omentum. A large pelvic-abdominal mass (~20 × 13 × 9 cm) originating from the right adnexa was identified (**Figure 1**). The mass was adherent to the right fallopian tube and partially attached to the greater omentum, extending from the pubic symphysis to the xiphoid process, with an intact surface and irregular solid consistency. The uterus was enlarged but otherwise unremarkable. A 3 cm cystic mass containing clear yellow fluid was present in the left ovary, along with a 1 cm mesosalpinx cyst on the same side. Small nodules (~0.3 cm) were noted in the left and right rectal depressions and the rectouterine pouch. After thorough consultation with the patient and her family regarding surgical options, risks, and benefits, a fertility-preserving approach was selected. The patient underwent transabdominal resection of the pelvic-abdominal mass, right adnexectomy, excision of left ovarian and mesosalpinx cysts, para-aortic and right pelvic lymphadenectomy, omentectomy, pelvic biopsies, and adhesiolysis. The mass was completely excised and submitted for pathological evaluation.

**Figure 2:**
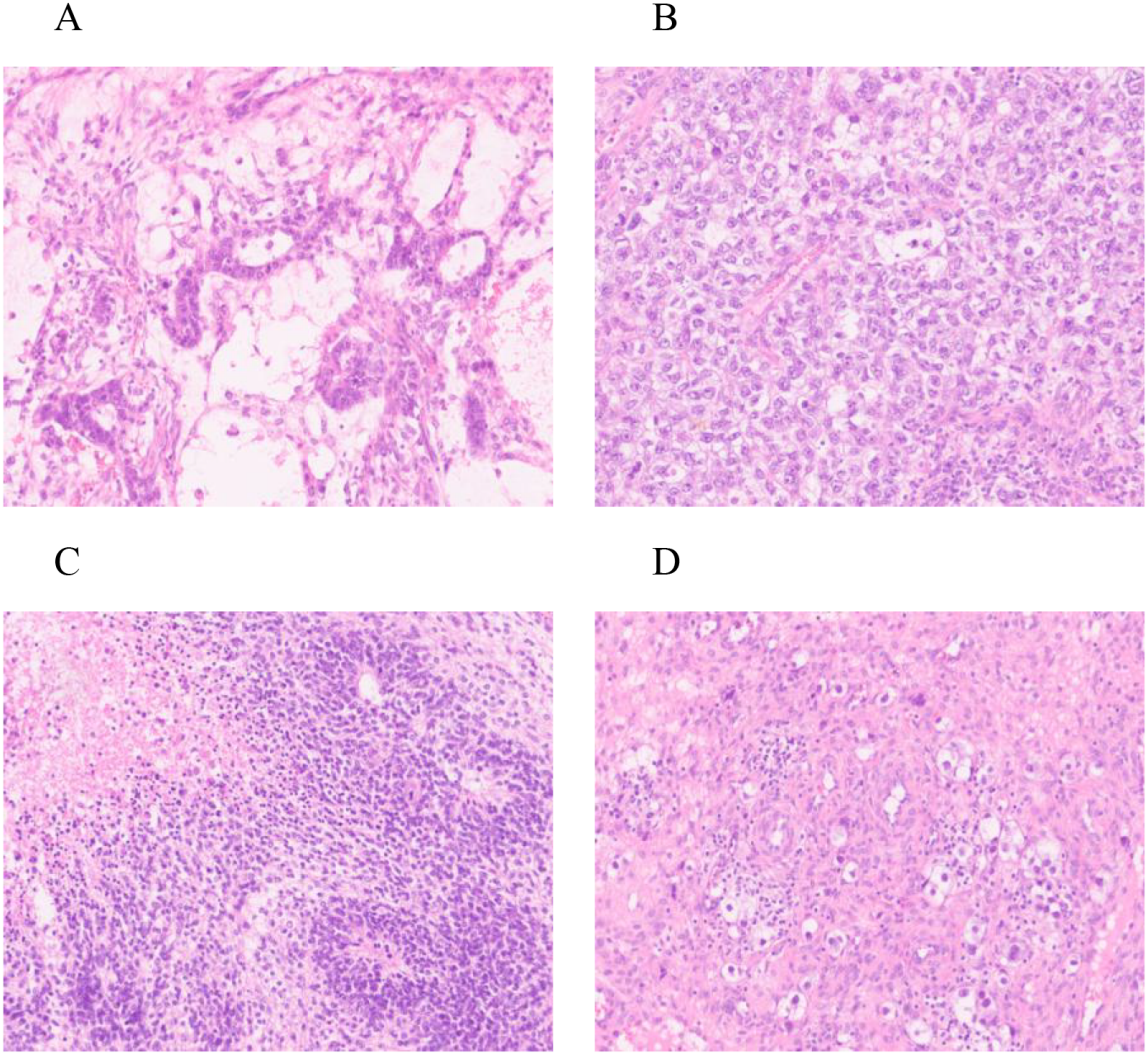
HE staining: Right ovary **(A)** yolk sac tumor * 200; **(B)** Embryonic cancer * 200; **(C)** Teratoma * 200; Left ovary **(D)** Dysgerminoma * 200.

**Figure 3:**
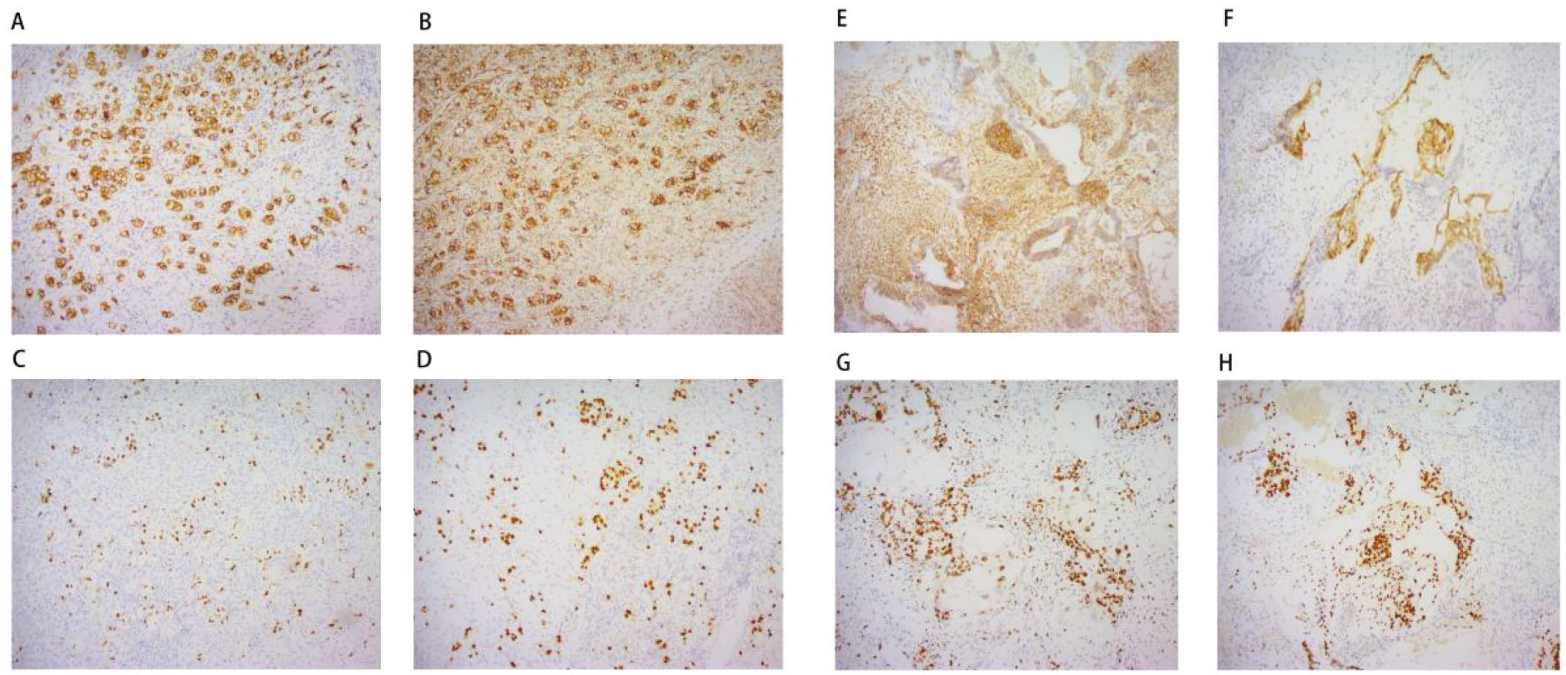
Immunohistochemistry: Left ovary **(A)** CD117 * 100; **(B)** D2 - 20 * 100; **(C)** Ki67 * 100; **(D)** SALL - 4 * 100; Right ovary **(E)** CD56 * 100; **(F)** Glypican-3 * 100; **(G)** Ki67 * 100; **(H)** SALL - 4 * 100;.

Histopathological examination (**Figure 2**) and immunohistochemistry confirmed a mixed germ cell tumor in the right adnexa, comprising approximately 60% yolk sac tumor(based on semiquantitative estimation from HE-stained sections by two independent pathologists), grade 3 immature teratoma, embryonal carcinoma, and a minor component of dysgerminoma. No metastases were identified in lymph nodes, omentum, or pelvic nodules. The left ovarian cyst was diagnosed as a hemorrhagic luteal cyst; however, dysgerminoma cells were detected in adjacent ovarian tissue, raising suspicion for contralateral dissemination. Immunohistochemical profiling (**Figure 3**) showed tumor cells positive for SALL - 4, partially positive for CD56 and Glypican-3, with Ki-67 indices ranging from 60% to 80%. Other markers showed variable expression: PAX - 8 negative, partial CD30 positivity, AE1/AE3 positive, and no evidence of TdT or WT - 1 expression. Ascitic fluid cytology was negative for malignancy, and no metastatic involvement was observed intraoperatively.

**Figure 4:**
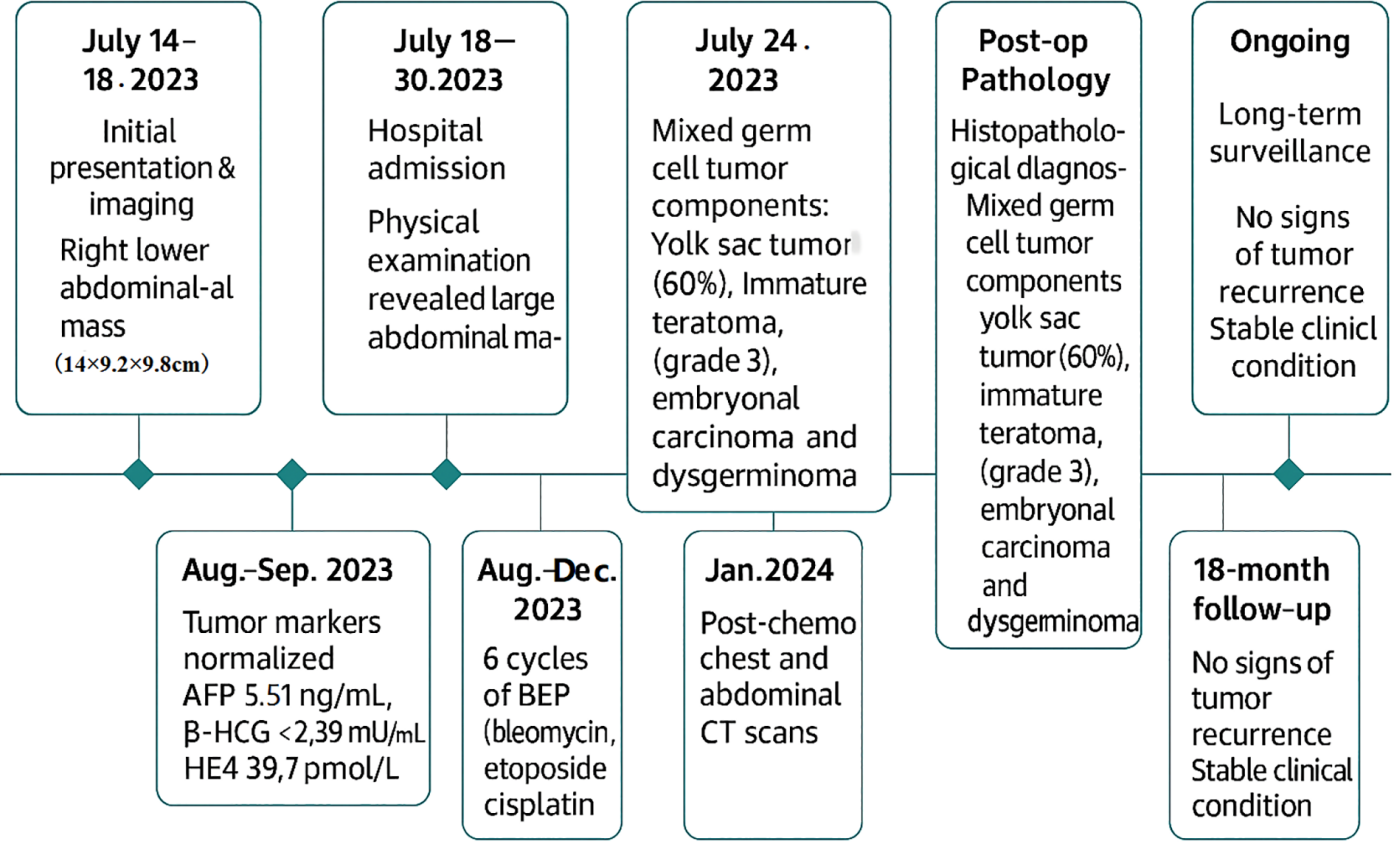
The timeline diagram for diagnosis and treatment follow-up.

Postoperatively, tumor markers normalized within two months (AFP 5.51 ng/mL, β-HCG <2.39 mIU/mL, HE4 39.7 pmol/L). The patient received six cycles of BEP chemotherapy (bleomycin 15 mg + etoposide 100 mg + cisplatin 30mg) every three weeks, which was well tolerated without significant adverse events. 18 months after surgery, no tumor recurrence had been observed. Long term follow-up is still ongoing. The timeline diagram for diagnosis and treatment follow-up was shown in **Figure 4**.

## Discussion

Mixed malignant ovarian germ cell tumors (MOGCTs) account for approximately 10 – 20% of all MOGCT cases (1). Their management remains challenging due to the need to balance oncologic treatment, fertility preservation, and prognosis (10). According to international data, 69.7% of patients are diagnosed at stage I, while stages II, III, and IV account for 7.5%, 18.2%, and 4.6%, respectively (11). In China, stage I cases account for 31.4% to 52.1% (12). Survival outcomes are influenced by age, stage, extent of surgery, and histological subtype. Platinum-based combination chemotherapy has significantly improved survival (13), though prognosis is poorer in patients over 45 years or with advanced disease. Early diagnosis and treatment are crucial to improve outcomes and preserve fertility in younger patients. Due to tumor heterogeneity and the potential for recurrence, clinical strategies must be carefully optimized. This study presents a retrospective analysis of a patient with a mixed MOGCT composed of four histological components and a contralateral dysgerminoma. We also summarize recent advances in diagnosis, treatment, and reproductive outcomes to provide reference for future clinical practice.

MOGCTs are typically large and fast-growing tumors. In adolescents, the most common symptoms are abdominal pain (87%) and abdominal masses (85%) (14). Around 10% of patients present with acute complications such as torsion, hemorrhage, or rupture, with mixed MOGCTs being more frequent in these cases (15).The patient in this case sought medical attention at an external hospital due to right abdominal pain. On July 14, 2023, ultrasound from the external hospital showed a mass of 14 * 9.2 * 9.8cm. Based on clinical symptoms, serum tumor markers—AFP, CA199, and LDH—were assessed. AFP is a glycogen protein, and in MOGCT (such as yolk sac tumors, embryonic cancers, and mixed tumors), AFP is usually greater than 1000 μ G/L. Especially for patients with simple yolk sac tumors, AFP levels can reach 10000 μ G/L or above (16). When tumor cells secreted hormones (such as β Human chorionic gonadotropin), or when hCG or serotonin was present, it could lead to endocrine manifestations such as irregular menstrual cycles and precocious puberty caused by hormone production by tumor cells. The differential diagnosis primarily included epithelial ovarian tumors, sex cord-stromal tumors, and benign ovarian masses. Epithelial ovarian tumors typically occur in older women and are often associated with elevated CA125 levels; however, in this case, CA125 was within normal limits, and the significantly elevated AFP and β-HCG supported the diagnosis of a germ cell tumor. Sex cord-stromal tumors may produce hormones causing endocrine symptoms, which were not observed in this patient. Imaging findings revealed a solid right adnexal mass and a cystic lesion in the left adnexa. Histopathology demonstrated multiple histological components consistent with a mixed malignant ovarian germ cell tumor. Immunohistochemical positivity for SALL - 4, Glypican-3, and CD30 further corroborated the diagnosis. Taken together, clinical presentation, imaging, tumor markers, and pathological features effectively excluded other malignancies and established the diagnosis of mixed malignant ovarian germ cell tumor. Currently, positron emission tomography (PET)/CT is more commonly used to monitor treatment response due to its lower accuracy and negative predictive value in distinguishing benign and malignant OGCT (17). Some new detection methods have also become more specific with the development of science, such as most dysgerminomas and yolk sac tumors, which can detect abnormalities in chromosome 12p through immunohistochemical staining and fluorescence *in situ* hybridization (FISH). SALL4 is a sensitive marker for germ cell tumors and is positive in most MOGCT subtypes (18). SOX2 expression is also seen in embryonal carcinoma and teratoma components (19). Advances in molecular markers may support earlier diagnosis and improved clinical management.

According to the European Society for Medical Oncology (ESMO) guidelines, fertility-sparing surgery should be prioritized in germ cell tumors (GCT) when feasible, especially in early-stage disease, with appropriate surveillance or adjuvant chemotherapy based on tumor characteristics and histology (20). The standard adjuvant regimen remains bleomycin, etoposide, and cisplatin (BEP) (21). In this case, no regional or distant metastases were found postoperatively, and the patient was staged as T1C N0 M0 (stage I). BEP chemotherapy (bleomycin 15 mg, etoposide 100 mg, cisplatin 30 mg for three days every three weeks) was initiated. Pathology revealed a large right adnexal mass composed of yolk sac tumor (approximately 60%), grade 3 immature teratoma, embryonal carcinoma, and focal dysgerminoma. The left ovary contained a hemorrhagic cyst with atypical clear cells, consistent with dysgerminoma. Although 3 – 4 cycles of BEP are typically recommended for MOGCT (22), the number of cycles may vary depending on residual disease. The NCCN guidelines recommend 6 cycles of adjuvant intravenous chemotherapy for stage I serous carcinoma, 3 cycles for other stage I epithelial cancers, and 6 cycles for stage II-IV epithelial diseases (regardless of tumor type) (23). We retained the left ovary of the patient. Considering the special condition of the patient’s condition and their demand for future fertility, after full communication with the patient, a total of six cycles of BEP chemotherapy were performed.

In addition to standard chemotherapy, targeted molecular therapies for MOGCT have been under investigation. Early data suggest that cyclin-dependent kinase 4/6 (CDK4/6) inhibitors may delay clinical progression in patients with unresectable mature teratomas (24). KIT tyrosine kinase, essential for normal germ cell development, is frequently mutated or overexpressed in ovarian germ cell tumors and testicular seminomas. This makes KIT a potential target for tyrosine kinase inhibitors such as imatinib to overcome cisplatin resistance (25). However, phase II trials of imatinib in unselected or KIT-positive germ cell tumors have shown limited efficacy, possibly due to activating mutations at the KIT kinase domain that confer resistance to blockade. A study by Yang et al. (26) conducted whole-exome and RNA sequencing on 41 tumor samples from 30 yolk sac tumor (YST) patients with varying responses to cisplatin. The analysis identified somatic mutations, copy number variations, microsatellite instability, and a unique mutation signature. Notably, overexpression of OVOL2 was associated with cisplatin resistance. These findings provide new insights into the molecular mechanisms of chemoresistance in YST and highlight potential targets for personalized therapy in MOGCT.

In some metastatic cancers, immunotherapy can produce sustained remission and provide significant long-term survival opportunities, but the role of immune checkpoint inhibitors in germ cell tumors still needs further research. In clinical trials of germ cell tumors, several immune oncology combinations are being tested (27). As recent studies have shown, gene modified T cells (CART cell therapy) with chimeric antigen receptors have shown encouraging clinical responses in various solid tumors and hematological tumors. Therefore, some studies have applied CART therapy to germ cell tumors. CD30 is a member of the tumor necrosis factor receptor superfamily. CD30 is a valuable diagnostic biomarker for detecting embryonic cancer in other types of germ cell tumors, but it plays a complex role in the development of embryonic cancer and further research is needed to understand its specific mechanisms and therapeutic applications in embryonic cancer (28). With the expansion of targeted biomarkers for CART cell therapy, more studies using CART to treat MOGCT are underway. However, the rarity and heterogeneity of tumors currently limit the development of CART cell therapy in MOGCT (29).

Malignant ovarian germ cell tumors (MOGCT) primarily affect young women, making fertility preservation an important consideration (30). For early-stage patients desiring fertility, uterus- and contralateral ovary-sparing surgery remains the gold standard (30). Standard surgical staging for ovarian malignancies includes omentectomy, peritoneal washings, biopsies, hysterectomy, bilateral salpingo-oophorectomy (BSO), and lymphadenectomy. However, due to the high chemosensitivity of MOGCT, the extent of staging surgery is still debated (31).Over the past two decades, surgical trends in MOGCT have shifted toward less extensive procedures. In a study of 2,238 patients (median age 21), only 12.4% underwent hysterectomy, one-third had omentectomy, and half had lymphadenectomy, with no significant survival difference observed (32). Fertility outcomes following conservative surgery are encouraging. Husaini et al. reported a 32% pregnancy rate, with 14 live births among 50 patients (33). Brewer et al. found that 71% maintained menstrual function during and after chemotherapy, and 35% achieved pregnancy (34).Current evidence does not support a survival benefit from more radical surgery in MOGCT (35). For this 22-year-old patient, fertility-sparing surgery (unilateral salpingectomy with preservation of the uterus and contralateral ovary) was performed. Follow-up remains uneventful. Adjuvant chemotherapy after fertility-preserving surgery is considered safe and may enhance reproductive outcomes (36).An Italian multicenter study identified pregnancy intention as the only predictor of live birth among 109 patients receiving fertility-preserving surgery and chemotherapy (30). A meta-analysis of 47 studies involving 2,189 patients also confirmed that most women retained normal reproductive function, with outcomes comparable to the general population (37). However, the optimal timing for pregnancy post-treatment remains unclear. Some studies recommend waiting at least 6 months after chemotherapy due to follicular maturation timelines (38).

New chemotherapy, targeted therapy, and immunotherapy offer hope for future MOGCT treatments, but BEP-based chemotherapy is still the main approach. For young patients, surgery should come first, with fertility-preserving options whenever possible. Women wanting to have children after chemotherapy should wait at least six months and get evaluated before trying to conceive. Because MOGCT is rare and data are limited, more case studies are needed to guide treatment. Mixed tumors affecting both ovaries make diagnosis and treatment harder. It is still necessary to conduct multicenter studies to optimize the diagnosis, treatment, and standardization of MOGCT. In addition, patients and their families should receive reproductive health counseling, and continuous long-term follow-up is crucial.

## Conclusions

Mixed malignant ovarian germ cell tumors (MOGCTs) are rare but aggressive tumors that primarily affect young women, making fertility preservation a key consideration in treatment planning. Our research report presents a case of bilateral MOGCT confirmed in our hospital, with the aim of providing some reference for clinical treatment of MOGCT. At present, the diagnosis and treatment plans for MOGCT vary in clinical practice. Fertility-sparing surgery combined with platinum-based chemotherapy remains the cornerstone of current management, offering favorable oncologic outcomes and good reproductive potential. Advances in molecular diagnostics and emerging targeted and immunotherapies hold promise for improving personalized treatment in the future. Due to the rarity and heterogeneity of MOGCTs, multicenter studies are needed to refine therapeutic strategies and optimize patient care. Long-term follow-up and reproductive counseling are essential components to support survivors’ quality of life.

## Data Availability

The original contributions presented in the study are included in the article/supplementary material. Further inquiries can be directed to the corresponding authors.
